# Giant cell tumour of the tendon sheath of the spine: clinical features and imaging findings

**DOI:** 10.1186/s13244-021-01025-2

**Published:** 2021-07-13

**Authors:** Piaoe Zeng, Annan Zhang, Le Song, Jianfang Liu, Huishu Yuan, Weifang Zhang

**Affiliations:** 1 Department of Radiology, Peking University Third Hospital, 49 North Garden Road, Haidian District, Beijing, 100191 China; 2 Department of Nuclear Medicine, Peking University Third Hospital, 49 North Garden Road, Haidian District, Beijing, 100191 China; 3 Key Laboratory of Carcinogenesis and Translational Research (Ministry of Education/Beijing), Department of Nuclear Medicine, Peking University Cancer Hospital & Institute, 52 Fucheng Road, Haidian District, Beijing, 100142 China

**Keywords:** Spine, Giant cell tumor of tendon sheath, Tomography (X-ray computed), Magnetic resonance imaging, Positron emission tomography-computed tomography

## Abstract

**Objectives:**

To review the clinical and imaging data of spinal giant cell tumour of the tendon sheath (GCTTS) to improve our understanding of the disease.

**Methods:**

The imaging findings, clinicopathological features and clinical outcomes of 14 patients with pathologically confirmed spinal GCTTS were analysed retrospectively.

**Results:**

All 14 patients had a single spinal lesion, including ten cervical vertebra lesions and four thoracic vertebra lesions. CT scan findings: The lesions showed osteolytic bone destruction and were centred on the facet joint, eroding the surrounding bone with a paravertebral soft tissue mass. MRI scan findings: all the lesions manifested predominantly as isointense or hypointense on T1-weighted imaging (T1WI). On T2-weighted imaging (T2WI), eight lesions were hypointense, and four were isointense. The remaining two lesions showed slight hyperintensity. The enhanced scans of eight lesions showed moderate to marked homogeneous or heterogeneous enhancement. PET/CT findings: Among the five patients who underwent PET/CT, three presented lesions with well-defined, sclerotic borders, and the uptake of ^18^F-FDG was markedly increased. One lesion showed an ill-defined border and an uneven increase in ^18^F-FDG uptake with an SUVmax value of 8.9. A recurrent lesion was only found on PET/CT 45 months after surgery and the SUVmax was 5.1.

**Conclusions:**

Spinal GCTTS is extremely rare. Osteolytic bone destruction in the area of the facet joint with a soft tissue mass and hypointensity on T2WI images are indicative of the spinal GCTTS. GCTTS shows high uptake of ^18^F-FDG, and PET/CT is helpful in detecting recurrent lesions.

## Key points

- Spinal giant cell tumour of the tendon sheath is extremely rare.
- Osteolytic bone destruction in the area of the facet joint with a soft tissue mass and hypointensity on T2WI images are indicative of spinal GCTTS.
- PET/CT is helpful in detecting recurrence of GCTTS.

## Introduction

Giant cell tumour of the tendon sheath (GCTTS) is a tumour like lesion that originates from synovial cells in the bursa, tendon sheath and joints with the possibility of malignancy. The most common site of this disease is in the tendon sheath of the hand and foot, followed by the large joints of the ankle, knee, hip, elbow, and shoulder. However, it rarely occurs in the spine. Preoperative diagnosis is challenging because of the low incidence and nonspecific symptoms of spinal GCTTS. In the present study, the clinical presentation, imaging findings, pathologic features, and clinical outcomes of fourteen patients with spinal GCTTS are presented, aiming to improve the awareness and diagnostic accuracy of the disease.

## Materials and methods

### Patients

The clinical and imaging data of fourteen patients with pathologically confirmed spinal GCTTS at our hospital from August 2007 to February 2020 were retrospectively analysed. The patient cohort included six male and eight female patients aged 13–49 years, with a median age of 34.5 years. In three patients, spinal GCTTS was found incidentally, presenting with no clinical symptoms. The other eleven patients had nerve compression symptoms, such as chest, back, neck and shoulder pain, accompanied by numbness and weakness of the upper limbs (Table 1). The lesions of all the patients were surgically resected or subjected to CT-guided biopsy, and the diagnosis was confirmed by pathology.

**Table 1:** General information of the 14 patients with spinal giant cell tumour of the tendon sheath (GCTTS)

Cases	Age (year)	Gender	Clinical manifestations	Benign/malignant	Initial treatment	Follow-up time (months)	Prognosis
1	32	Female	Back pain with left upper numbness for 2 years	Benign	Gross total resection	157	Recurrence-free survival
2	45	Male	Back pain	Benign	Gross total resection	117	Recurrence after 45 months
3	28	Male	Neck pain more than 1 year, right upper limb radiating pain and right-hand numbness and weakness for 6 months	Benign	Cytoreductive surgery and postoperative radiotherapy	76	Progression-free survival
4	32	Female	Neck pain for 2 years	Benign	Cytoreductive surgery and postoperative radiotherapy	74	Progression-free survival
5	47	Female	Neck and shoulder pain for 1 year, right upper limb numbness and weakness for 40 days	Malignant	Gross total resection and postoperative radiotherapy	53	Recurrence-free survival
6	32	Male	Thoracic, back and shoulder pain for 2 years	Benign	Gross total resection	Loss to follow-up	–
7	13	Female	Left upper limb numbness for 1.5 months	Benign	No treatment	Loss to follow-up	–
8	49	Male	Numbness or weakness of limbs for 6 years	Benign	Cytoreductive surgery and postoperative radiotherapy	36	Progression-free survival
9	44	Male	Thoracic and back pain for 6 months	Benign	Radiotherapy alone	36	Progression-free survival
10	38	Female	Asymptomatic	Benign	Gross total resection and postoperative radiotherapy	28	Recurrence-free survival
11	36	Female	Back pain with numbness in both hands for 1 year	Benign	Gross total resection	28	Recurrence-free survival
12	17	Female	Neck pain for 3 years, upper limbs numbness for 9 months, right lower limb weakness for 2 weeks	Benign	Gross total resection	8	Recurrence-free survival
13	31	Female	Asymptomatic	Benign	No treatment	Loss to follow-up	–
14	39	Male	Asymptomatic	Benign	No treatment	30	Progression-free survival

### Imaging examination

All the patients underwent CT and MRI scans, and eight of them received contrast-enhanced CT and/or MRI scans. Five patients underwent PET/CT imaging, in four of them the referring clinicians wanted to exclude malignant transformation, and in one patient recurrence was suspected.

The CT scans were performed using a Siemens Somatom Definition Flash dual-source CT device (Siemens, Erlangen, Germany) or a GE Discovery 750 HD CT device (GE Medical Systems, Chalfont St. Giles, UK) at a tube voltage of 120 kV, a tube current of 280 mA, a pitch of 1.0, and a slice thickness of 3–4 mm. For enhanced scanning, a non-ionic contrast agent (Iopamiro; 350 mg I/ml) was injected via the elbow vein using a high-pressure injection system at a dose of 2 ml/kg and a rate of 3.0 ml/s.

The MRI scans were performed using a GE Discovery MR750 3.0 T (GE Healthcare, Piscataway, NJ, USA), a GE Signa HDxT 3.0 T (GE Healthcare, Sunnyvale, CA, USA), or a Siemens Magnetom Trio Tim 3.0 T device (Siemens, Erlangen, Germany) for scans with a slice thickness of 3.0 mm and an intersection gap of 0.3 mm. The scanning sequence included axial T2-weighted imaging (T2WI), sagittal T2WI, sagittal T1-weighted imaging (T1WI), and fat-suppression T2WI. The sequence scan parameters were as follows: T1WI: repetition time (TR), 360–600 ms and echo time (TE), 10–20 ms; T2WI: TR, 2200–4400 ms and TE, 100–120 ms. For contrast enhancement, gadopentetate dimeglumine(0.5 mmol/ml; Beilu Pharmaceutical, Beijing, China) was injected via an elbow vein at a dose of 2.0 ml/kg and an injection rate of 2.0 ml/s. Fat-suppression axial, sagittal, and coronal T1WI was performed after injection with the following parameters: TR 600–700 ms and TE 11 ms.

For PET/CT, a 52-ring Siemens Biograph 64 True Point PET/CT device was also used. ^18^F-FDG was provided by the Institute of Isotope Research in China Academic of Atomic Energy with > 90% radiochemical purity. After fasting for more than 6 h, each patient was administered 5.55 MBq/kg (0.15 mCi/kg) ^18^F-FDG intravenously and imaged with routine PET/CT after 60 min. The CT scan range was from the skull base to the upper femur, with a matrix of 512 × 512. Next, PET images were collected with a matrix of 168 × 168 for 5–7 beds (2–2.5 min per bed). Thereafter, with the patient maintaining the same position, a deep inspiratory HRCT scan was performed using a 64 × 1.25-mm detector array, with a pitch of 0.53 and 1.25-mm collimation (120 kVp and 100 mAs). PET images were reconstructed by ordered subset expectation maximisation (OSEM). Subsequently, image fusion and evaluation were performed using a MedEx PET/CT image and information system.

### CT-guided core needle biopsy

Thirteen of fourteen patients underwent CT-guided core needle biopsy. The patients were placed in a lateral or prone position, sterilised and anaesthetised layer by layer with 1% lidocaine. The coaxial biopsy needle was used as the trocar. An automatic biopsy needle was used to obtain pathological specimens, which were fixed by 10% formalin. Following biopsy, the patients were rescanned to detect any complications, if present.

### Image post-processing and analysis

CT, MRI, and PET/CT images were analysed by two experienced imaging doctors (with 8 and 4 years of experience in bone tumour imaging, respectively) separately, and then the final diagnosis was decided by group discussion when the opinions were inconsistent. The location, bone destruction, margin, size, density (compared with muscle)/signal (compared with the spinal cord signal), cortical bone continuity, sclerotic margin, surrounding soft tissue mass, and degree of enhancement were observed. A region of interest (ROI) was drawn along the border of the lesion at the slice with the highest level of ^18^F-FDG uptake observed in the lesion, and the maximum standard uptake value (SUVmax) of the lesion was automatically measured by the computer.

### Treatment and outcome

Among the fourteen patients, one received only radiotherapy, three received no treatment, and the remaining ten underwent surgery. Seven patients underwent gross total resection of the tumour, two of whom underwent additional postoperative radiotherapy. Three patients underwent cytoreductive surgery and postoperative radiotherapy (Table 1). Re-examinations were performed 3, 6 and 12 months post-operatively. In the absence of recurrence, follow-up was performed once every 6–12 months. Follow-up observations included patient symptoms and X-ray, CT and/or MRI scans.

## Results

### Imaging manifestations

Ten lesions were in the cervical vertebrae, and four were in the thoracic vertebrae. GCTTSs were centred on the facet joint, involving the vertebral body, vertebral arch and spinous process as a soft tissue mass. The maximal diameters ranged from 15.7 to 66.4 mm, with an average of 35.6 mm (Table 2).

**Table 2:** Imaging manifestations of the 14 patients with spinal giant cell tumour of the tendon sheath (GCTTS)

Cases	Segment	Location	CT findings					MRI findings		Enhancement	SUV_max_	Size (mm)
			Soft Tissue Mass	Border	Density	Cortical bone Discontinuous	Sclerotic margin	T1WI signal	T2WI signal			
1	C5-7	Left vertebral plate, left part of vertebral body and spinous process	(+)	Well	Heterogeneous	(+)	(+)	Hypointense, with internal slight hyperintensity	Mostly hypointense with internal hyperintensity	–	–	32.4 × 30.9 × 35.0
2	T9 (primary)	Left vertebral arch, transverse process and left part of vertebral body	(+)	Ill	Heterogeneous	(+)	(+)	Mostly hypointense	Mostly hypointense	Obviously heterogeneous		65.6 × 33.1 × 61.4
	T11 (recurrent)	Lesion unable to be clearly observed due to severe metal artefacts on CT and MRI images									5.1	13 × 8 × 7*
3	C4-5	Right transverse process and pedicle of vertebral arch of C4; vertebral body, right transverse process and spinous process of C5	(+)	Ill	Heterogeneous	(+)	(−)	Isointense and hyperintense	Mostly slightly hyperintense with internal hypointensity	Obviously heterogeneous		47.8 × 46.1 × 43.4
4	C1-2	Anterior arch and left lateral mass of C1; left vertebral arch, transverse process and left part of vertebral body of C2	(+)	Wel	Homogeneous, isodense	(+)	(+)	Isointense	Hypointense	–		45.7 × 36.9 × 40.
5	C4-6	Most of vertebral body, bilateral transverse process and vertebral arch	(+)	Ill	Homogeneous, isodense	(+)	(−)	Isointense with internal hypointensity	Mostly hypointense with internal hyperintensity	–	8.9	48.6 × 36.0 × 37.7
6	T1-2	Left vertebral arch, transverse process and articular process; left part of vertebral body	(+)	Well	Homogeneous, isodense	(−)	(+)	Isointense	Isointense	Obviously homogeneous	15.5	31.6 × 27.2 × 18.0
7	C5	Left lamina of vertebral arch	(+)	Well	Heterogeneous	(−)	(−)	Isointense	Isointense	Obviously homogeneous		17.6 × 12.4 × 9.0
8	C1-2	Left lateral mass, posterior arch of C1 and left vertebral arch, transverse process of C2	(+)	Ill	Homogeneous, isodense	(+)	(+)	Hypointense	Hypointense	–		66.4 × 52.5 × 51.1
9	T3	Right vertebral arch, transverse process and articular process	(+)	Well	Homogeneous, isodense	(−)	(−)	Isointense and hypointense	Slightly hyperintense	Obviously homogeneous		28.5 × 21.6 × 24.6
10	C1	Posterior arch	(+)	Well	Heterogeneous	(+)	(+)	Isointense	Hypointense	Moderately homogeneous		23.9 × 21.6 × 17.6
11	C4-5	Right transverse process, pedicle of vertebral arch and articular process	(+)	Wel	Heterogeneous	(+)	(+)	Hypointense	Hypointense	Obviously heterogeneous		31.1 × 19.0 × 17.6
12	C6	Right vertebral arch	(+)	Well	Homogeneous, isodense	(+)	(−)	Isointense	Isointense	–		20.4 × 13.5 × 21.3
13	C6-7	Right transverse foramen, pedicle of vertebral arch and articular process	(+)	Well	Homogeneous, isodense	(−)	(+)	Isointense	Hypointense	Moderatelyhomogeneous	15.5	20.7 × 19.6 × 17.0
14	T8	Left costotransverse articulation	(−)	Well	Homogeneous, isodense	(+)	(+)	Isointensity	Isointensity	–	15.3	11. × 10.4 × 15.7

*The size of the lesion was measured on the high uptake region on PET/CT examination

#### CT findings

**Fig. 1:**
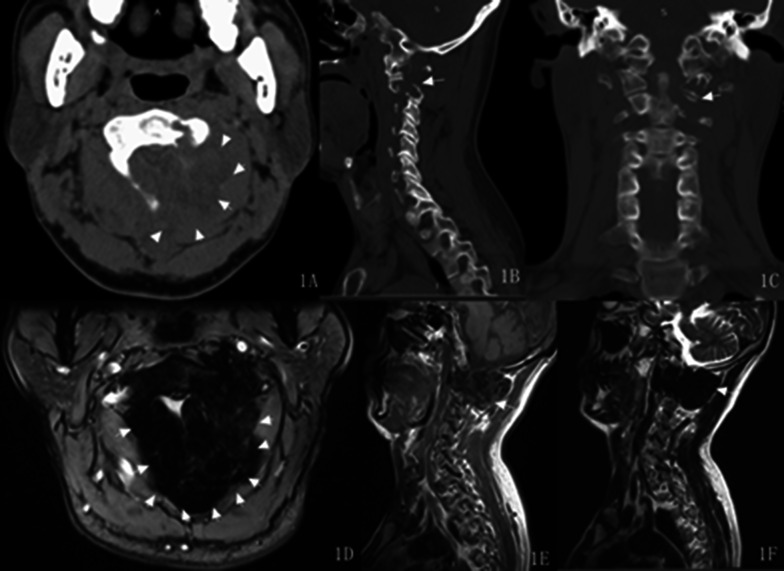
A 49-year-old male with benign GCTTS. The lesion, centred on the facet joint of the C1-2 vertebra, was expansive and osteolytic bone destruction with cortical discontinuity (**A**–**C**, arrow). The tumour appeared mainly hypointense on T1WI (**E**, arrow) and hypointense on T2WI (**D**, **F**, arrow)

Most lesions, centred on the facet joints, showed osteolytic bone destruction with cortical discontinuity (Fig. 1A–C). 10 out of 14 lesions showed well-defined borders while in 4 they were ill-defined. A sclerotic margin was observed in eight lesions. Compared with that of the surrounding muscles, six lesions showed heterogeneous density, and the other eight showed homogeneous density. Six patients underwent an enhanced scan; one lesion showed marked heterogeneous enhancement, three showed marked homogeneous enhancement and two showed moderate homogeneous enhancement. The CT findings are summarised in Table 2.

#### MRI findings

**Fig. 2:**
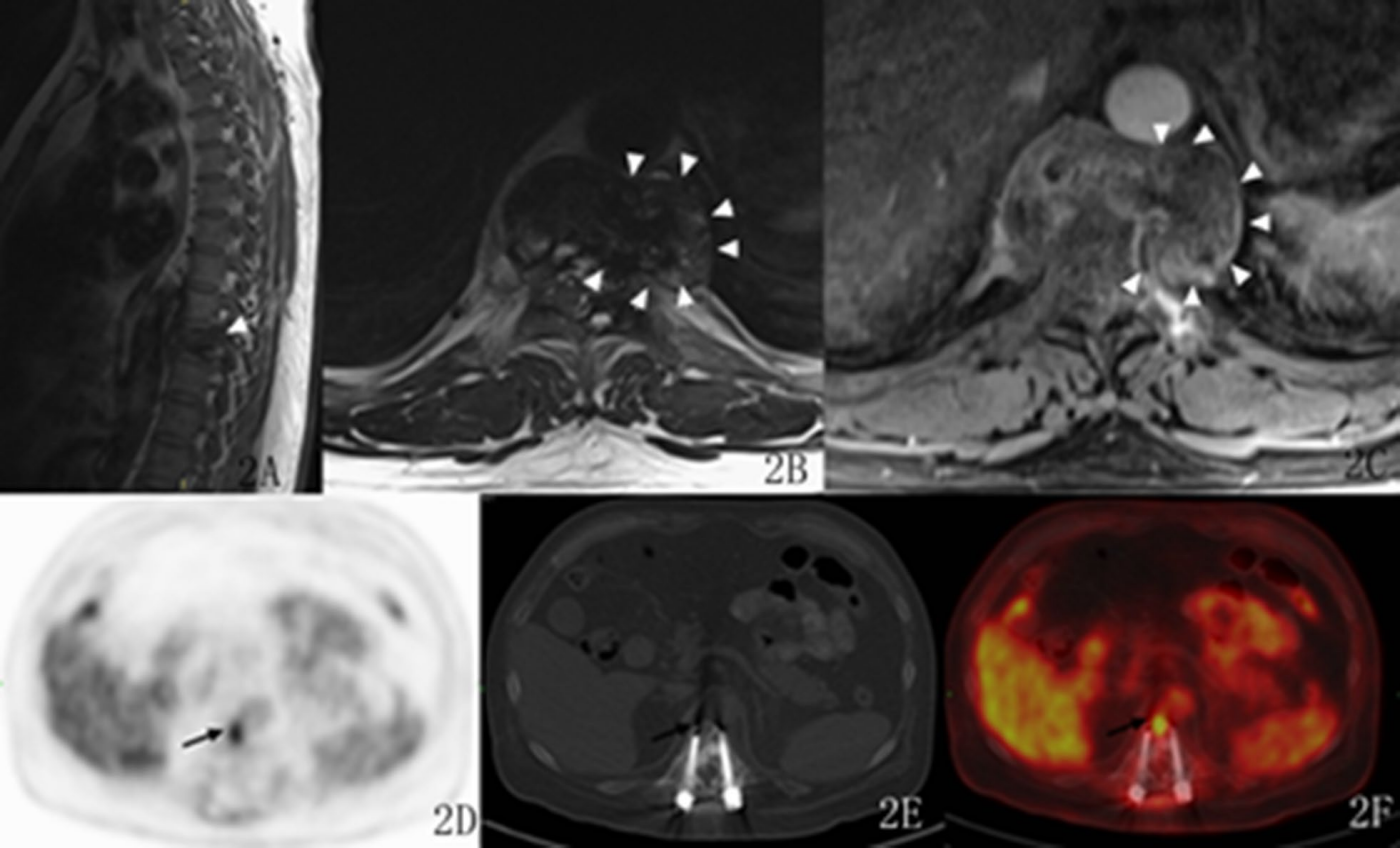
A 45-year-old male with benign GCTTS. The lesion was hypointense on T1WI (**A**, arrow) and mostly hypointense on T2WI in the T9 vertebra (**B**, arrows). Enhanced MRI scans revealed heterogeneous enhancement (**C**, arrows). Recurrence was suspected after surgery, and PET showed high ^18^F-FDG uptake in the vertebral body (**D**, arrow). The lesion was blurred on CT because of the interference from artefacts (**E**, arrow), but the fusion image clearly showed the high ^18^F-FDG-uptake lesion at the anterior edge of the T11 vertebra (**F** arrow)

**Fig. 3:**
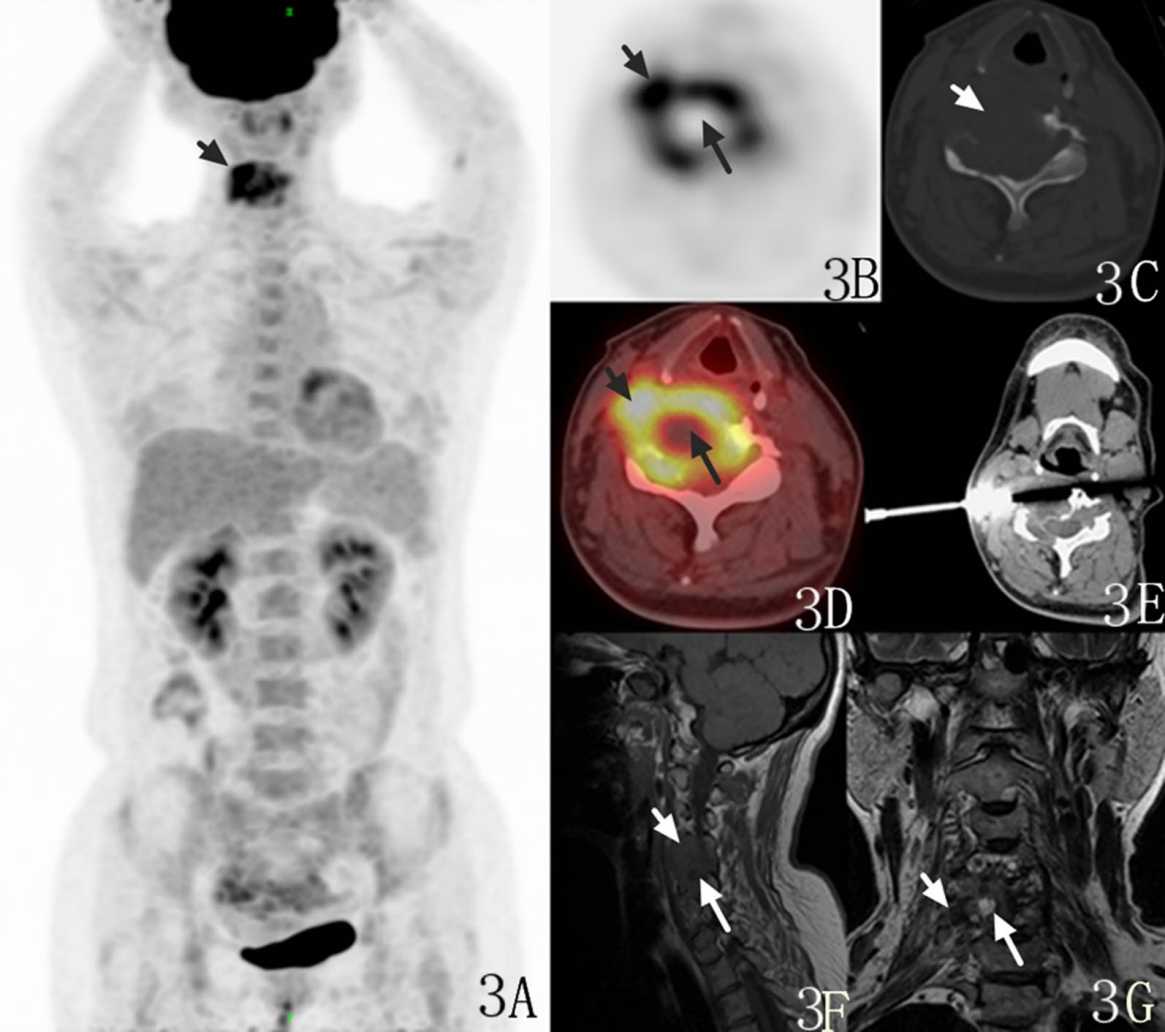
A 47-year-old female with malignant GCTTS. PET/CT maximum intensity projection (MIP) images revealed increased but heterogeneous uptake of FDG in the C4-6 vertebra (**A**, arrow), and no other lesions were found in any part of the body. The spinal lesion shows heterogeneous FDG uptake with central deficiency (**B**, **D**, long arrow) but higher uptake in the periphery (**B**, **D**, short arrow), with an SUVmax value of 8.9. CT showed irregular osteolytic bone destruction (**C**, arrow). CT-guided biopsy was performed on the hypermetabolic area of the lesion (**E**). The lesion showed signal hypointensity on sagittal T1WI image with unclear margin (**F**, short and long arrows) and hyperintensity in the central region but hypointensity in the periphery on coronal T2WI images (**G**, short and long arrows)

The tumour signal appeared mainly isointense or hypointense on T1WI compared with the spinal cord signal in all the patients (Figs. 1E, 2A). On T2WI, eight lesions appeared hypointense (Fig. 1D, F). Among these eight lesions, two displayed a cystic region in the centre that was hyperintense on T2WI (Figs. 2B, 3G) and hypointense on T1WI (Fig. 3F), and one displayed signal hyperintensity on both T2WI and T1WI inside. Four lesions appeared isointense on T2WI, and the remaining two lesions featured slight signal hyperintensity on T2WI. Four out of seven patients showed moderate to marked homogeneous enhancement, while three disclosed marked heterogeneous enhancement (Fig. 2C). The imaging manifestations are detailed in Table 2.

#### PET/CT findings

Among the five patients who underwent PET/CT examination, except for local bone lesions, abnormal hypermetabolic lesions were not observed in any part of the body (Fig. 3A). The lesions of 3 patients showed expansive and osteolytic bone destruction on CT, and the borders were smooth, with sclerotic margins. The ^18^F-FDG uptake was evenly increased, with SUVmax values of 15.1, 15.5, and 15.3. One lesion was malignant and showed irregular osteolytic bone destruction with a paravertebral soft tissue mass, which had indistinct borders (Fig. 3C), and an uneven increase in ^18^F-FDG uptake with an SUVmax value of 8.9 (Fig. 3B).

CT-guided core needle biopsy (17G × 6.8 cm coaxial biopsy needle as the cannula, and 18G × 10 cm BioPince automatic biopsy needle) was performed on the PET/CT hypermetabolic lesion, which was pathologically confirmed to be a malignant GCTTS (Fig. 3D, E). PET/CT was performed for another patient suspected of recurrence after surgery, revealing high uptake in the vertebral body (Fig. 2D). Because of pronounced metal artefacts MRI and CT were degraded and a clear diagnosis could not be made, whereas the high 18F-FDGuptake allowed for assessing the recurrency. (Fig. 2F).

### Pathological manifestation

**Fig. 4:**
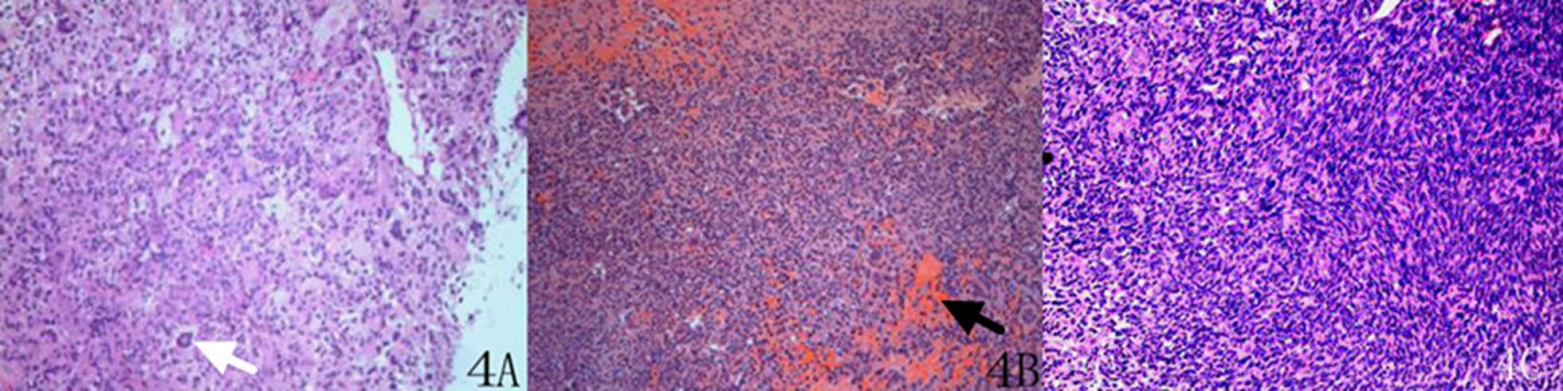
**A** Benign GCTTS showed mixed inflammatory cells, foam cells and haemosiderin, accompanied by multinucleated giant cells (white arrow) and fibrosis. The cells had no obvious atypia, HE 10 × 10. **B** Malignant GCTTS showed multifocal cystic changes, necrosis and haemorrhage (black arrow), HE 10 × 10. **C** Malignant GCTTS comprised densely arranged splindle mononuclear cells, showing enlarged nuclei and an increased mitotic count. Multinucleated giant cells could be identified among the mononuclear components, HE 10 × 40

Thirteen lesions were benign GCTTSs, and one was a malignant GCTTS. In benign GCTTSs, micronuclear hyperplasia was observed in the microscopic examination, demonstrating some mixed inflammatory cells, foam cells and haemosiderin, accompanied by several multinucleated giant cells and fibrosis (Fig. 4A). The cells had no obvious atypia. The malignant lesion showed dense and uniformly round cells. The cells showed atypia and a sarcoma-like structure. Multifocal cystic changes, necrosis and haemorrhage were also observed in the lesion (Fig. 4B).

### Prognosis

Three patients were lost to follow-up. The follow-up time was 8–157 months, with an average of 58 months. Only one patient exhibited recurrence after 45 months of follow-up. The remaining thirteen patients had no recurrence or progression. (Table 1).

## Discussion

GCTTS is a type of soft tissue tumour that originates from the tendon sheath and synovium. However, it can also manifest as a lesion with bone involvement. GCTTS is classified as a fibrohistiocytic tumour based on the 2013 soft tissue tumour classification and can be divided into the localised type and diffuse type according to its growth pattern [1]. The very uncommon malignant tenosynovial giant cell tumour is defined by the coexistence of a benign tenosynovial giant cell tumour with overtly malignant areas or by the recurrence of a typical giant cell tumour as a sarcoma. In our study, one case was diagnosed with malignant GCTTS. Malignant GCTTSs are extremely rare and fewer than 40 have been reported [2]. GCTTS occurs most frequently close to peripheral bone, especially adjacent to large joints such as the knee, hip, and elbow. GCTTS is rare in the spine, and there are only few case reports. To our knowledge, our study is the largest case cohort of spinal GCTTS from a single centre. The incidence of spinal GCTTS in female patient was slightly higher than that in male patient in our study. The female to male ratio was 1.33:1, which is roughly concordant with that in previous studies [3, 4].

A systematic review of 69 cases of spinal GCTTS [3] found that the median age at disease diagnosis was 38.5 ± 17.9 years, which is consistent with the median age of 34.5 years in this study. The clinical manifestations of spinal GCTTSs are nonspecific, and patients may be asymptomatic or suffer from radicular pain and numbness, depending on the size and location of the tumour. The patient’s symptoms may be related to compression of the nerve root by a soft spinal mass. Most spinal GCTTS lesions occur in the cervical spine (53%), followed by the thoracic (27%) and lumbar regions (20%) [5]. In the present study, 10 lesions were located in the cervical spine, and 4 were located in the thoracic spine. Furthermore, all spinal GCTTSs were in close proximity to the facet joint and eroded the adjacent bone (and involved the vertebral body in 5 cases), suggesting that the lesions originated from the synovial tissue of the facet joints. GCTTS shows osteolytic bone destruction with sclerotic margins associated with a nodular soft tissue mass and rarely presents with calcification [6].

MRI is highly useful in imaging bone and soft tissue tumours and enables to suggests the composition of the neoplasms. In our study, spinal GCTTS generally showed signal isointensity or hypointensity relative to the spinal cord signal on T1WI. The signals on T2WI were mostly isointense to hypointense because of the paramagnetic effect of haemosiderin. On gradient-echo sequences, this finding is more obvious because of blooming artifacts [7–9]. The signals on T2WI can also be variable, depending on mass components, which may have variable haemosiderin, liquid, lipid, and fibrous tissue contents and haemorrhage. In the present study, GCTTSs showed moderate to marked enhancement, probably due to the proliferative capillaries within the collagenous stroma within the lesion [10].

^18^F-FDG PET/CT imaging of spinal GCTTS has been infrequently reported [11–14]. In previous studies, GCTTS showed a high ^18^F-FDG uptake [11–14]. All the lesions in our study showed high ^18^F-FDG uptake, consistent with previous report findings. The reason may be mainly related to the pathological components of GCTTS, which are rich in monocytes, foam cells, multinucleated giant cells and inflammatory cells, all of which highly express glucose transporters GLUT-1 and GLUT-3 and hexokinase-2 (HK-2) [15, 16]. These cells can fully take up glucose in a low-sugar environment, resulting in high uptake by GCTTS. Because of this high uptake, the value of PET/CT in diagnosing GCTTS among primary spinal tumours is limited. However, PET/CT is critical to detect recurrence. In our study, one patient who underwent gross total resection of the lesion developed back pain 45 months after the surgery. Due to severe metal artifacts, CT and MRI examination of the thoracic vertebra were unable to precisely demonstrate lesion recurrence. PET/CT examination revealed a high uptake of ^18^F-FDG in the T11 vertebrae, and the patient underwent radiotherapy.

GCTTS must be differentiated from various benign and malignant spinal tumours. Because tumours of the spine are most commonly metastatic, differentiation between GCTTS and metastatic tumours should be a priority, in which the clinical history and PET/CT can be useful. Additionally, spinal GCTTS must be differentiated from spinal giant cell tumour of bone (GCTB) and schwannoma. GCTBs also contain multinucleated giant cells, monocytes and haemosiderin deposits, thus, the MRI and PET/CT imaging findings can be similar to those of GCTTS. However, GCTB of the spine often occurs in the sacrum and mostly affects the vertebral body instead of the posterior elements [17, 18]. Schwannoma often manifests as a dumbbell-shaped mass and enlarges the intervertebral foramen without bone destruction. Obvious cystic changes and haemorrhagic areas are more common in GCTB and schwannoma [19], contributing to the differential diagnosis of spinal GCTTS.

## Conclusion

GCTTS of the spine is extremely rare but must be differentiated from other benign and malignant tumours. Osteolytic bone destruction in the area of the facet joint with a soft tissue mass and hypointensity on T2WI images are indicative of the spinal GCTTS. PET/CT is helpful in monitoring recurrent lesions.

## Data Availability

The analysed and/or used datasets in this study can be obtained on reasonable request to the corresponding author.

## Abbreviations

GCTB: giant cell tumour of bone
GCTTS: giant cell tumour of the tendon sheath
ROI: region of interest
SUVmax: maximum standard uptake value
T1WI: T1-weighted imaging
T2WI: T2-weighted imaging
TE: echo time
TR: repetition time
